# Alfieri stitch for temporary severe functional mitral regurgitation after aortic valve replacement

**DOI:** 10.1186/s40792-017-0410-3

**Published:** 2018-01-08

**Authors:** Yoshihisa Morimoto, Takaki Sugimoto

**Affiliations:** 1 Department of Cardiovascular Surgery, Ako City Hospital, 1090 Nakahiro, Ako, Hyogo 678-0232 Japan; 2 Department of Cardiovascular Surgery, Awaji Medical Center, Hyogo, Japan

**Keywords:** Alfieri edge-to-edge stitch, Functional mitral regurgitation, Weaning from cardiopulmonary bypass

## Abstract

The outcome in functional mitral regurgitation after aortic valve replacement is unclear. A frail 82-year-old woman with severe aortic valve regurgitation and mild to moderate functional mitral valve regurgitation (NYHA functional class III) was referred to our clinic. In consideration of her frail condition, aortic valve replacement without mitral surgery was performed. She had hemodynamic instability and difficulty to wean off cardiopulmonary bypass caused by severe functional mitral valve regurgitation with left ventricular dilatation. A central Alfieri edge-to-edge stitch was placed between the anatomical middle of the two leaflets of the mitral valve after reinstitution of cardiopulmonary bypass. This eliminated the mitral regurgitation, which enabled successful separation from cardiopulmonary bypass.

**Electronic supplementary material:**

The online version of this article (10.1186/s40792-017-0410-3) contains supplementary material, which is available to authorized users.

## Background

Although mitral regurgitation severity may decrease after isolated aortic valve replacement, it may not improve and may even worsen in a substantial proportion of patients, and a subsequent mitral valve procedure is associated with increased operative risk in such cases [1]. The optimal surgical approach to functional mitral regurgitation remains uncertain.

Described by Alfieri in a report by Fucci and colleagues [2], the edge-to-edge technique facilitates mitral valve repair in a variety of situations. The edge-to-edge technique can significantly improve functional mitral regurgitation [3].

We report a case in which edge-to-edge repair was used to treat increased functional mitral regurgitation after aortic valve replacement.

## Case presentation

**Fig. 1:**
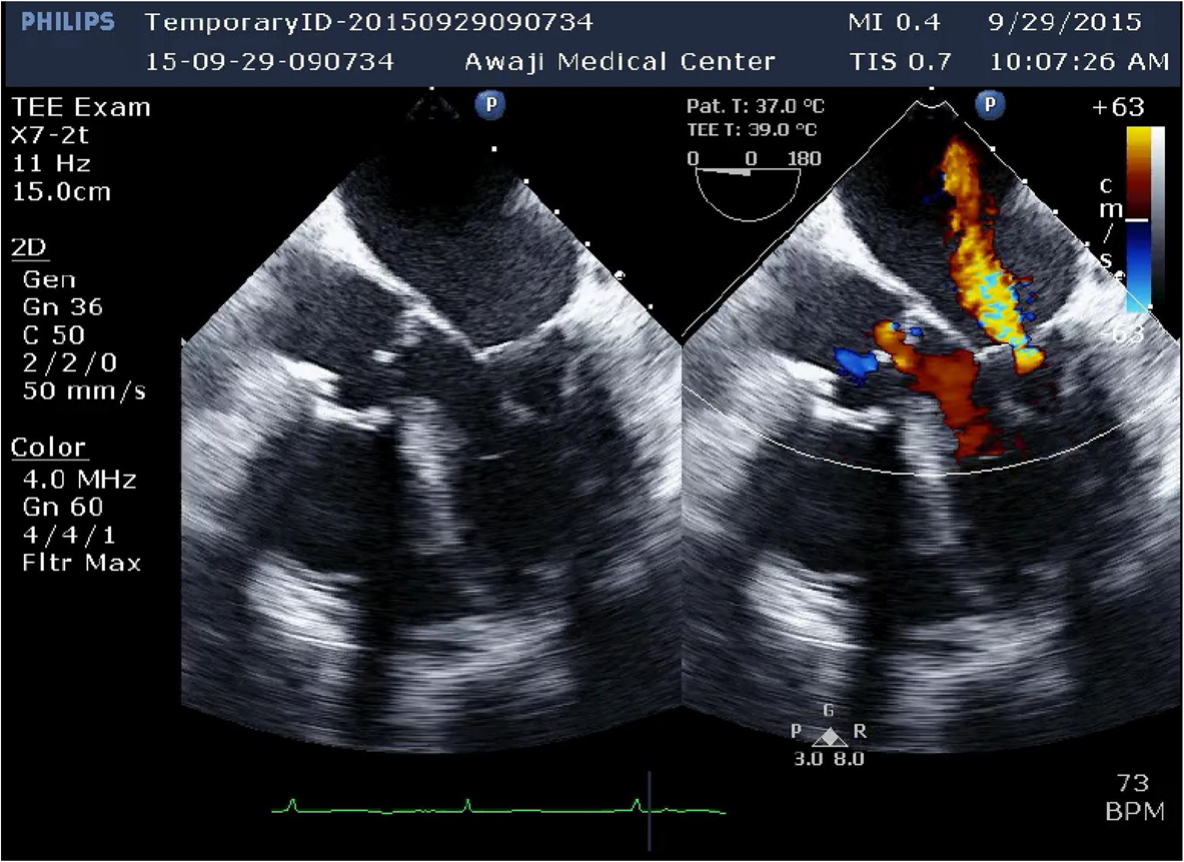
Preoperative transesophageal echocardiography images showing mitral valve and a color Doppler image showing mild to moderate functional mitral regurgitation

**Fig. 2:**
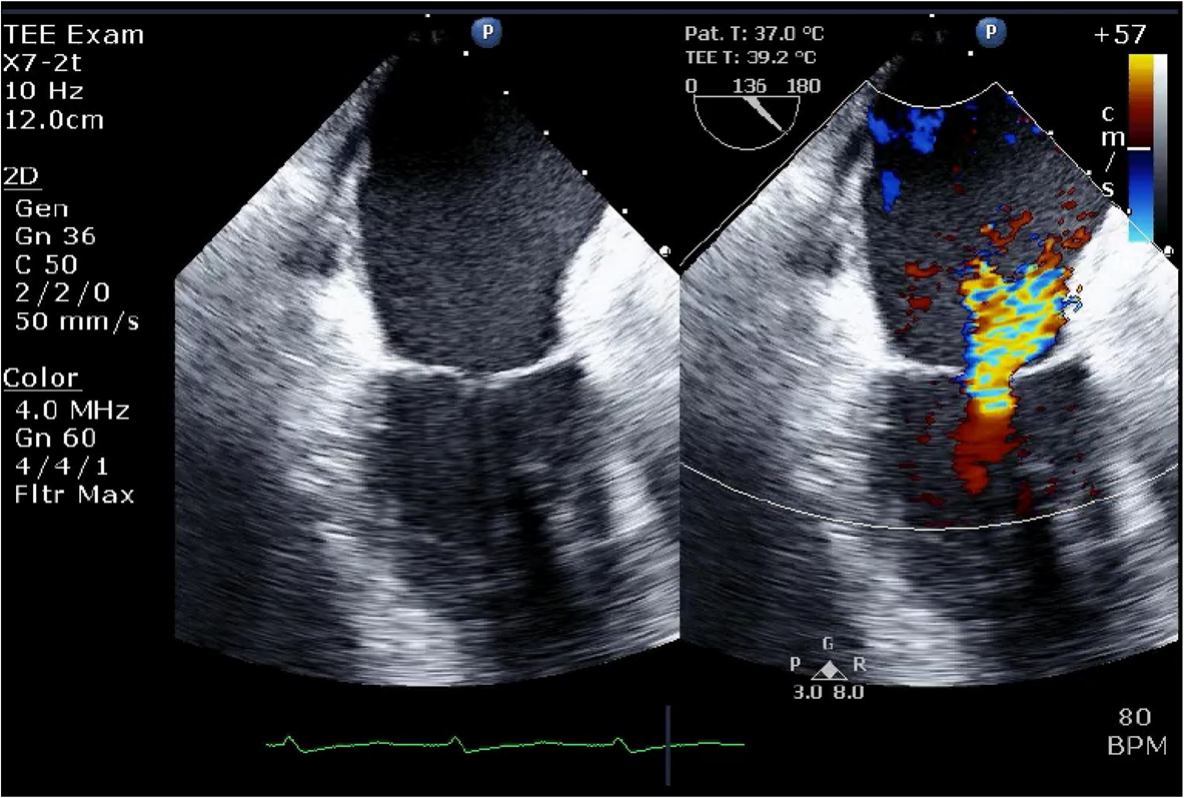
Intraoperative transesophageal echocardiography images after aortic valve surgery, showing a color Doppler image showing severe functional mitral regurgitation caused by left ventricular dilatation

A frail 82-year-old woman was referred to our clinic with shortness of breath and palpitations. Her echocardiogram demonstrated left ventricular ejection fraction of 65%, severe aortic valve regurgitation, mild to moderate functional mitral valve regurgitation (Carpentier type IIIb mechanism, effective regurgitant orifice area [EROA] 0.1 cm2, regurgitant volume [RV] 13 mL, color area of MR 6.3 cm2, mitral annulus 27 mm, Fig. 1, Additional file 1: Video S1), and increased systolic pulmonary artery pressures 50 mmHg. In consideration of her frail condition and our expectation of difficult mitral valve exposure due to her very bent back, aortic valve replacement (Mitroflow 21 mm) without mitral surgery was performed. The aortic cross-clamp time was 92 min. The transesophageal echocardiogram revealed left ventricular ejection fraction of 55% and severe functional mitral regurgitation caused by left ventricular dilatation on weaning from cardiopulmonary bypass. The etiology of MR was identified as asymmetric posterior leaflet tethering (Carpentier type IIIb mechanism, EROA 0.2 cm^2^, RV 29 mL, color area of MR 11.3 cm^2^, mitral annulus 32 mm, Fig. 2, Additional file 2: Video S2), a result of temporary left ventricular dysfunction. She had hemodynamic instability and difficulty to wean off cardiopulmonary bypass. **Additional file 1: Video S1.** Preopertative transesophageal echocardiography images showing mitral valve and a color Doppler image showing mild to moderate functional mitral regurgitation. (MP4 1044 kb)**Additional file 2: Video S2.** Intraoperative transesophageal echocardiography images after aortic valve surgery, showing a color Doppler image showing severe functional mitral regurgitation caused by left ventricular dilatation. (MP4 1063 kb)

The aorta was cross clamped again and the heart was arrested. As expected, it is difficult to expose her mitral valve due to her very bent back and after aortic valve replacement. There was no abnormality of the mitral apparatus. A central edge-to-edge Alfieri stitch (mattress 4–0 braided suture) was placed between the anatomical middle of the two leaflets of the mitral valve. The second aortic cross-clamp time was 46 min.

**Fig. 3:**
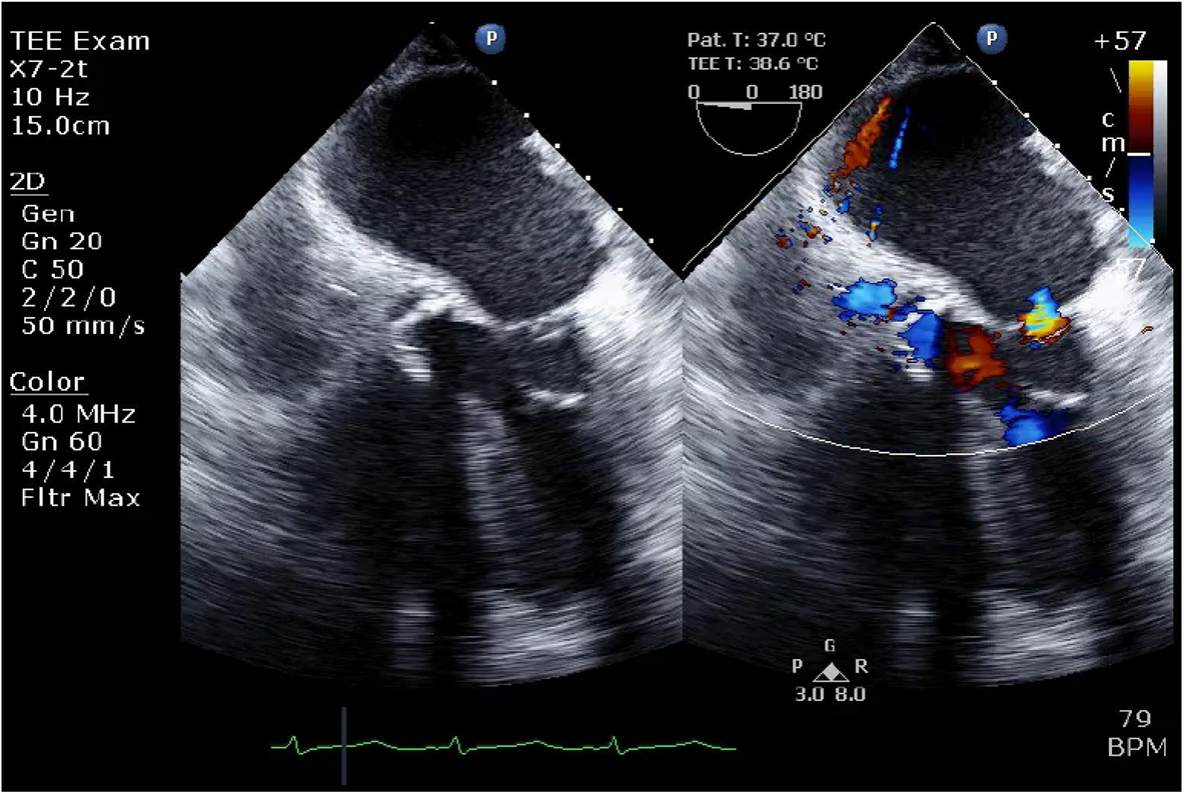
Intraoperative transesophageal echocardiography images after Alfieri stitch was performed, showing a color Doppler image showing mild to moderate residual mitral regurgitation

Transesophageal echocardiogram demonstrated mild mitral regurgitation (Carpentier type IIIb mechanism, EROA 0.1 cm2, RV 10 mL, color area of MR 4.3 cm2, mitral annulus 32 mm, Fig. 3, Additional file 3: Video S3). The patient was then successfully weaned from cardiopulmonary bypass. The patient made an uneventful recovery and was discharged home on post-operative day 14.**Additional file 3: Video S3.** Intraoperative transesophageal echocardiography images after alieri stitch was performed, showing a color Doppler image showing mild to moderate residual mitral regurgitation. (MP4 1050 kb)

## Discussion and conclusion

The outcome in functional mitral regurgitation patients after aortic valve replacement is unclear. Although uncommon in other circumstances, in this patient, the constellation of dilated ventricular cavity and left ventricular dysfunction caused by aortic valve replacement resulted in hemodynamically significant functional mitral regurgitation upon weaning from cardiopulmonary bypass.

Successful weaning from cardiopulmonary bypass required direct surgical treatment to eliminate severe functional mitral regurgitation. We were reluctant to perform mitral valve replacement or repair due to the unstable clinical condition of the patient.

The Alfieri edge-to-edge stitch technique is a rapid and effective way of mitral valve repair and is associated with satisfactory clinical outcomes in several diseases. Placement of a central Alfieri edge-to-edge stitch eliminated severe functional mitral valve regurgitation and restored hemodynamic stability with a functional rather than an anatomical repair. The Alfieri edge-to-edge stitch technique facilitates rapid and effective treatment of severe functional mitral regurgitation and may be of benefit in challenging critical situations and difficult mitral valve exposure.

Previous literature presented about severe mitral annulus calcification and/or systolic anterior movement of anterior mitral leaflet [4]. We report here that we can repair intraoperative functional MR due to temporary LV dysfunction with Alfieri stitch.

However, data on the technical aspects of the Alfieri edge-to-edge stitch technique are insufficient in this critical patient population and further prospective clinical studies are needed to determine its durability.

In conclusion, the Alfieri edge-to-edge stitch technique showed promising short-term results in a critical patient with functional mitral regurgitation after aortic valve replacement. This technique is much more rapid than other techniques and may be considered as an alternative technique for severe functional mitral regurgitation after aortic valve replacement. Cardiac surgeons must be vigilant in observing any deterioration of functional mitral regurgitation after aortic valve replacement.

## Abbreviations

EROA: Effective regurgitant orifice area
MR: Mitral regurgitation
RV: Regurgitant volume

## References

[1] Alghamdi AA, Elmistekawy EM, Singh SK, Latter DA (2010). Is concomitant surgery for moderate functional mitral regurgitation indicated during aortic valve replacement for aortic stenosis? A systematic review and evidence-based recommendations. J Card Surg.

[2] Fucci C, Sandrelli L, Pardini A, Torracca L, Ferrari M, Alfieri O (1995). Improved results with mitral valve repair using new surgical techniques. Eur J Cardiothorac Surg.

[3] De Bonis M, Lapenna E, La Canna G, Ficarra E, Pagliaro M (2005). Mitral valve repair for functional mitral regurgitation in end-stage dilated Cardiomyopathy role of the “edge-to-edge” technique. Circulation.

[4] Sherlock KE, Muthuswamy G, Basu R, Mitchell IM (2011). The Alfieri stitch: the advantages for mitral valve repair in difficult circumstances. J Card Surg.

